# Genetic and bioprocess engineering to improve squalene production in *Yarrowia lipolytica*

**DOI:** 10.1016/j.biortech.2020.123991

**Published:** 2020-12

**Authors:** Huan Liu, Fang Wang, Li Deng, Peng Xu

**Affiliations:** a Department of Chemical, Biochemical and Environmental Engineering, University of Maryland Baltimore County, Baltimore, MD 21250, China; b College of Life Science and Technology, Beijing University of Chemical Technology, Beijing, China

**Keywords:** Oleaginous yeast, Metabolic engineering, Mevalonate pathway, Squalene, Mannitol cycle

## Abstract

Squalene is the precursor for triterpene-based natural products and steroids-based drugs. It has been widely used as pharmaceutical intermediates and personal care products. The aim of this work is to test the feasibility of engineering *Yarrowia lipolytica* as a potential host for squalene production. The bottleneck of the pathway was removed by overexpressing native HMG-CoA (3-hydroxy-3-methylglutaryl-CoA) reductase. With the recycling of NADPH from the mannitol cycle, the engineered strain produced about 180.3 mg/L and 188.2 mg/L squalene from glucose or acetate minimal media. By optimizing the C/N ratio, controlling the media pH and mitigating acetyl-CoA flux competition from lipogenesis, the engineered strain produced 502.7 mg/L squalene, a 28-fold increase over the parental strain (17.2 mg/L). This work may serve as a baseline to harness *Y. lipolytica* as an oleaginous cell factory for sustainable production of squalene or terpenoids-based chemicals and natural products.

## Highlights

• Enhancement of endogenous HMG-CoA reductase removed pathway bottlenecks.
• Optimization of NADPH and acetyl-CoA supply improved squalene titer.
• C/N ratio and media pH were optimized to improve squalene accumulation.
• A 28-fold increase of squalene titer was achieved in an engineered *Y. lipolytica*.

## 1 Introduction

*Yarrowia lipolytica* is an industrial oleaginous yeast that has been extensively engineered to synthesize lipophilic compounds, including lipids (Qiao et al., 2017), oleochemicals (Xu et al., 2016), carotenoids (Larroude et al., 2018), terpenoids (Jin et al., 2019) and aromatic polyketides (Czajka et al., 2018, Lv et al., 2019b) *et al.* The lipogeneity of this yeast makes it a superior host to produce chemicals that are derived from acetyl-CoA, malonyl-CoA, 3-hydroxy-3-methylglutaryl coenzyme A (HMG-CoA) and NADPHs (Gu et al., 2020, Ma et al., 2020). The compartmentalization of oil droplets into lipid bodies provides a hydrophobic environment to sequester many lipid-related compounds and mitigate the toxicity issues associated with lipophilic membrane damages. In addition, the ease of genetic manipulation, substrate flexibility and robust growth present us tremendous opportunity to upgrade low-value renewable feedstocks to high-value compounds. It has also been recognized as a ‘generally regarded as safe’ (GRAS) organism (Groenewald et al., 2014) in the food and nutraceutical industry. A large collection of customized genetic toolboxes, including YaliBricks gene assembly (Wong et al., 2017), CRISPR-Cas9 (Bae et al., 2020, Larroude et al., 2020) or CRISPR-Cpf1 (Yang et al., 2020) genome editing, Cre-LoxP-based iterative chromosomal integrations (Lv et al., 2019a), transposon-based mutagenesis (Wagner et al., 2018) and Golden-gate cloning (Egermeier et al., 2019, Larroude et al., 2019), enabled us to rapidly modify its genome and evaluate many metabolic events to explore the catalytic diversity of this yeast beyond its regular portfolio of fatty acids, fatty alcohols, biofuels *et al*. Recent metabolic engineering effort in this yeast has allowed us to access more specialized secondary metabolites with pharmaceutical values, including sesquiterpenes (Marsafari & Xu, 2020), triterpenoids (Jin et al., 2019) and flavonoids (Lv et al., 2019b, Palmer et al., 2020) *et al*.

Isoprenoids are a large group of natural products with diverse biological functions. An estimation of >70,000 isoprenoids, ranging from monoterpenes, sesquiterpenes, diterpenes and triterpenes have been discovered from nature (Moser & Pichler, 2019). Isoprenoids play a major role in maintaining membrane homeostasis, protein prenylation for subcellular targeting (Palsuledesai & Distefano, 2015), signal transduction, the deployment of plant defense pathways, and controlling the transcriptional activity of sterol-responsive-element-binding-proteins (SREBPs) (Shimano, 2001). The yeast-based mevalonate (MVA) pathway starts with acetyl-CoA condensation reactions, proceeds through the reduction of intermediate HMG-CoA *via* HMG-CoA reductase, which is the rate-limiting step and the molecular target to design many statins-related anti-cholesterol drugs (Xie & Tang, 2007). The universal five-carbon precursors isopentenyl diphosphate (IPP) and dimethylallyl diphosphate (DMAPP), derived from mevalonate, are condensed to make the farnesyl pyrophosphate (FPP), which later can be diversified to many sesquiterpenes or triterpenes. Squalene is a 30-carbon triterpene hydrocarbon synthesized from the condensation of two FPPs, which serve as the gateway molecule for all triterpenoids with tens of thousands of structural variations. Squalene possesses strong antioxidant and anti-inflammatory activity and is widely used in the cosmetic industry as skin-compatible super-lubricant and hydration protectors (Spanova & Daum, 2011). Squalene emulsions were used as efficient adjuvants to enhance the immune response of certain vaccines (Spanova & Daum, 2011). Squalene is primarily sourced from shark liver, which poses significant ecological or ethical concerns related with shark-hunting. Reconstitution of squalene pathway in microbes may provide an alternative route to sustainably produce squalene from renewable feedstocks. A number of metabolic engineering studies have set the effort to engineer bacteria or bakers’ yeast to produce squalene, with improved yield and process efficiency from glucose (Fagundes et al., 2019, Huang et al., 2018). A recent work identified that yeast peroxisome may serves as a dynamic depot to store squalene up to 350 mg/g dry cell weight (Liu et al., 2020), despite the highly oxidative nature of peroxisome. In this work, we report the systematic optimization and engineering of the endogenous mevalonate pathway in *Y. lipolytica* for efficient synthesis of squalene from simple synthetic media. The bottlenecks of the mevalonate pathway were identified and alternative reducing equivalents (NADPH) pathways to improve squalene production were discovered. The engineered strain produced up to 502.7 mg/L of squalene in shake flask. This work may set a foundation for us to explore oleaginous yeast as a chassis for cost-efficient production of squalene and triterpenoids in a long-term run.

## 2 Methods and materials

### 2.1 Strains and culture conditions

*Escherichia coli* NEB 5α high efficiency competent cells were obtained from NEB (New England Biolabs inc.) for plasmid construction, preparation, propagation and storage. The *Y. lipolytica* wild type strain W29 was purchased from ATCC (ATCC20460). The auxotrophic Po1g (Leu−) was obtained from Yeastern Biotech Company (Taipei, Taiwan). All strains and plasmids are listed in Table 1.

**Table 1:** Strains and plasmids used in this study.

**Plasmid or strain**	**Relevant properties or genotype**	**Source**
**Plasmids**		
PYLXP’	pYaliA1 vector backbone with leucine marker and Ampicillin resistance gene	Xu Peng et al
PYLXP’-SQS	PYLXP’ carrying SQS from *Y. lipolytica*	This study
PYLXP’-SQS-ylHMG1	PYLXP’ carrying SQS and HMG1 from *Y. lipolytica*	This study
PYLXP’-SQS-ylt495HMG1	PYLXP’ carrying SQS and truncated HMG1 from *Y. lipolytica*	This study
PYLXP’-SQS-SctHMG1	PYLXP’ carrying SQS from *Y. lipolytica*, truncated HMG1 from *S. cerevisiae*	This study
PYLXP’-SQS-SpHMG1	PYLXP’ carrying SQS from *Y. lipolytica*, HMG1 from *Silicibacter pomeroyi*	This study
PYLXP’-SQS-ylHMG1-ylErg8	PYLXP’ carrying SQS, Erg8 and HMG1 from *Y. lipolytica*	This study
PYLXP’-SQS-ylHMG1-ylErg10	PYLXP’ carrying SQS, Erg10 and HMG1 from *Y. lipolytica*	This study
PYLXP’-SQS-ylHMG1-ylErg12	PYLXP’ carrying SQS, Erg12 and HMG1 from *Y. lipolytica*	This study
PYLXP’-SQS-ylHMG1-ylErg20	PYLXP’ carrying SQS, Erg20 and HMG1 from *Y. lipolytica*	This study
PYLXP’-SQS-ylHMG1-ScErg8	PYLXP’ carrying SQS and HMG1 from *Y. lipolytica*, Erg8 from *S. cerevisiae*	This study
PYLXP’-SQS-ylHMG1-ScErg12	PYLXP’ carrying SQS and HMG1 from *Y. lipolytica*, Erg12 from *S. cerevisiae*	This study
PYLXP’-SQS-ylHMG1-ylGPS	PYLXP’ carrying SQS, GPS and HMG1 from *Y. lipolytica*	This study
PYLXP’-SQS-ylHMG1-ylMAE	PYLXP’ carrying SQS, MAE and HMG1 from *Y. lipolytica*	This study
PYLXP’-SQS-ylHMG1-ylUGA2	PYLXP’ carrying SQS, UGA2 and HMG1 from *Y. lipolytica*	This study
PYLXP’-SQS-ylHMG1-ylGND2	PYLXP’ carrying SQS, GND2 and HMG1 from *Y. lipolytica*	This study
PYLXP’-SQS-ylHMG1-ylIDP2	PYLXP’ carrying SQS, IDP2 and HMG1 from *Y. lipolytica*	This study
PYLXP’-SQS-ylHMG1-ylMnDH1	PYLXP’ carrying SQS, MnDH1 and HMG1 from *Y. lipolytica*	This study
PYLXP’-SQS-ylHMG1-ylMnDH2	PYLXP’ carrying SQS, MnDH2 and HMG1 from *Y. lipolytica*	This study
PYLXP’-SQS-ylHMG1-ylPDC1-ylALD3	PYLXP’ carrying SQS, PDC1, ylALD3 and HMG1 from *Y. lipolytica*	This study
PYLXP’-SQS-ylHMG1-ylPDC1-ylALD4	PYLXP’ carrying SQS, PDC1, ylALD4 and HMG1 from *Y. lipolytica*	This study
PYLXP’-SQS-ylHMG1-ScPDC1-ScADH	PYLXP’ carrying SQS and HMG1 from *Y. lipolytica*, PDC1 and ADH from *S. cerevisiae*	This study
PYLXP’-SQS-ylHMG1-ScPDC1-EcPuuC	PYLXP’ carrying SQS and HMG1 from *Y. lipolytica*, PDC1 from *S. cerevisiae*, PuuC from *E.coli.*	This study
PYLXP’-SQS-ylHMG1-ylACL1	PYLXP’ carrying SQS, ACL1 and HMG1 from *Y. lipolytica*	This study
PYLXP’-SQS-ylHMG1-ylACL2	PYLXP’ carrying SQS, ACL2 and HMG1 from *Y. lipolytica*	This study
**Strains**		
*E. coli NEB5α*	fhuA2 Δ(argF-lacZ)U169 phoA glnV44 Φ80 Δ(lacZ)M15 gyrA96 recA1 relA1 endA1 thi-1 hsdR17	New England Biolabs
*Y. lipolytica po1g*	matA, xpr2-332, axp-2, leu2-270	Madzak C et al
*HLYaliS01*	*Y. lipolytica po1g with vector PYLXP’-SQS-ylHMG*	This study
*HLYaliS02*	*Y. lipolytica po1g with vector PYLXP’-SQS-ylHMG1-ylMnDH2*	This study
*HLYaliS03*	*Y. lipolytica po1g with vecto PYLXP’-SQS-ylHMG1-ylACL2*	This study
*HLYaliS04*	*Y. lipolytica po1g with vecto PYLXP’-SQS-ylHMG1- ylMnDH2-ylACL2*	This study

LB broth or agar plate with 100 µg/mL ampicillin was used to cultivate *E. coli* strains. Yeast rich medium (YPD) was prepared with 20 g/L Bacto peptone (Difco), 10 g/L yeast extract (Difco), and 20 g/L glucose (Sigma-Aldrich), and supplemented with 15 g/L Bacto agar (Difco) for solid plates. YNB seed medium was made with 1.7 g/L yeast nitrogen base (without amino acids and ammonium sulfate) (Difco), 5 g/L ammonium sulfate (Sigma-Aldrich), 0.69 g/L CSM-Leu (Sunrise Science Products, Inc.) and 20 g/L glucose. Selective YNB plates were made with YNB media supplemented with 15 g/L Bacto agar (Difco). In fermentation process, YNB fermentation medium with glucose as substrate and carbon/nitrogen molar ratio of 80:1 was made with 1.7 g/L yeast nitrogen base (without amino acids and ammonium sulfate), 1.1 g/L ammonium sulfate, 0.69 g/L CSM-Leu and 40 g/L glucose. YNB fermentation medium with sodium acetate as substrate and carbon/nitrogen molar ratio 80:1 was made with 1.7 g/L yeast nitrogen base (without amino acids and ammonium sulfate), 0.825 g/L ammonium sulfate, 0.69 g/L CSM-Leu, 41 g/L sodium acetate. Glacial acetic acid was purchased from Sigma-Aldrich.

Phosphoric buffer solution (PBS) with pH 6.0 was made with 0.2 M Na_2_HPO_4_ and 0.2 M Na_2_HPO_4_, which was used to replace water to make YNB- glucose-PBS fermentation medium. Bromocresol purple was a pH-sensitive indicator which could change its color with the pH from 5.2 to 7.0 (El-Ashgar et al., 2012) and 40 mg/L bromocresol purple was added into fermentation medium to indicate pH variation. The pH of medium was regulated to 6.0 by 6.0 M HCl in the fermentation process. The components in glucose-YNB media with C/N ratio 60:1, 40:1, 20:1, 10:1 were as same as them in C/N ratio 80:1 except the content of ammonium sulfate changed to 1.47 g/L, 2.2 g/L, 4.4 g/L, 8.8 g/L respectively, to explore the effect of C/N ratio on squalene accumulation. And 1 mg/L cerulenin solution prepared with dimethylsulfoxide (DMSO) was added into fermentation medium to inhibit precursor (fatty acids biosynthesis) competing pathway. All the experiments in our work were triplicates and mean data with standard deviations were reported.

### 2.2 Genetic transformation of *Y. lipolytica*

All plasmids constructed were transformed into the *Y. lipolytica* host strain Po1g (Leu−) using the lithium acetate/single-strand carrier DNA/PEG method (Chen et al., 1997). Single fresh *Y. lipolytica* colonies were picked from YNB selective plates and inoculated into YNB seed media, which were grown at 30 °C for 48 h. For tube test, 100 μL seed cultures were inoculated into 5 mL fermentation media in 50 mL tube. 600 μL seed cultures were inoculated into 30 mL fermentation media in 250 mL shake flasks with 250 rpm and 30 °C. Time series samples were taken for analyzing biomass, sugar content, and squalene titer.

### 2.3 Analytical methods

Four OD units of liquid yeast cell was harvested and subsequently was pelleted by centrifugation at 13,200 × g for 5 min. Water was completely withdrawn and 500 μL 0.5 M sodium methoxide (sodium hydroxide dissolved in pure methanol) was used to resuspend the cell pellet. The mixture was kept at room temperature with shaking for 2 h at 1,200 rpm with a high-duty vortex to allow complete saponification of lipids and extraction of squalene. Then 400 μL hexane was added to extract squalene. The mixture was vortexed at room temperature for 10 min and hexane phase was directly injected to gas chromatography-FID (GC-FID) for squalene analysis. Gas chromatography–flame ionization detector (GC-FID) system (Agilent 7820A) equipped with HP-5 column (30 m × 320 μm × 0.25 μm) was used to detect squalene, using helium as the carrier gas with a linear velocity of 2 mL/min. The column temperature profile was 175 ℃ for 3 min, 20 ℃/min ramping to 200 ℃ and holding for 3 min, and then 20 ℃/min ramping to 260 ℃ and holding for 4 min.

The cell growth was monitored by measuring the optical density at 600 nm (OD_600_) with a UV–vis spectrophotometer that could also be converted to dry cell weight (DCW) according to the calibration curve DCW:OD_600_ = 0.33:1 (g/L). The fermentation broth was centrifuged at 13,200 × g for 5 min and the supernatant was used for analyzing the concentration of glucose, mannitol, and acetic acid by HPLC with a refractive index detector and Supelcogel ^TM^ Carbohydrate column. The column was eluted with 10 mM H_2_SO_4_ at a column temperature of 50 ℃, a refractive index detector temperature of 50 ℃, and a flow rate of 0.4 mL/min.

### 2.4 Plasmid and pathway construction

All restriction enzymes were purchased from Fisher FastDigest enzymes. Plasmid miniprep, PCR clean-up, and gel DNA recoveries were using Zyppy and Zymoclean kits purchased from Zymo research. All the genes were PCR-amplified with the primers from genomic DNA of *Y. lipolytica*, *S. cerevisiae*, *E. coli*, *B. subtilis, Aspergillus nidulans*, respectively (Supplementary data and Table 1). All these genes were inserted into downstream of the *Y. lipolytica* TEF-intron promoter in the pYLXP’ vector backbone (Wong et al., 2017) at the SnaBI and KpnI site *via* Gibson assembly (Gibson et al., 2009). Upon sequence verification by Genewiz, the restriction enzyme *Avr*II, *Nhe*I, *Not*I, *Cla*I and *Sal*I (Fermentas, Thermo Fisher Scientific) were used to digest these vectors, and the donor DNA fragments were gel purified and assembled into the recipient vector containing previous pathway components in compliance with the YaliBricks subcloning protocol (Wong et al., 2019). All assembled plasmids were verified by gel digestion and were subsequently transformed into the *Y. lipolytica* host strain Po1g (Leu − ) using the lithium acetate/single-strand carrier DNA/PEG method (Chen et al., 1997). In chromosomal integration process, pYLXP’ vector assembled with functional genes was linearized by restriction enzyme NotI (Fermentas, Thermo Fisher Scientific). The linear fragment was transformed into the *Y. lipolytica* host strain Po1g (Leu − ) and cultivated on CSM-Leu minimal media (agar plate) for colony screening. The screened colony was later cultivated in YPD media and genomic DNA was extracted with Wizard genomic kits (Promega). Then the genomic samples were used as template for PCR verification of the integrated gene with gene-specific primers.

## 3 Results and discussions

### 3.1 Debottlenecking mevalonate pathway for squalene production

**Fig. 1:**
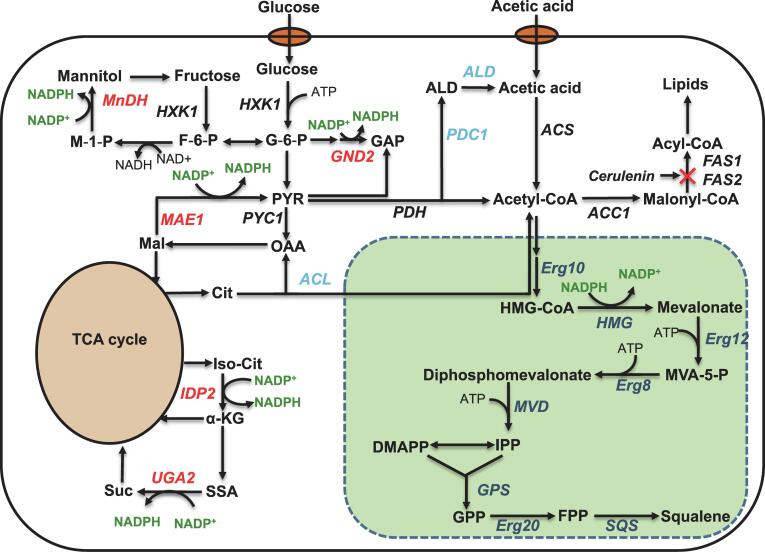
Metabolic pathway for squalene synthesis in oleaginous yeast. MnDH, mannitol dehydrogenase; HXK, hexokinase; MAE1, malic enzyme; ACL2, ATP citrate lyase; IDP2, cytosolic NADP-specific isocitrate dehydrogenase; UGA2, succinate semialdehyde dehydrogenase; PYC1, pyruvate carboxylase; PDC1, pyruvate decarboxylase; ALD, aldehyde dehydrogenase; PDH, pyruvate dehydrogenase complex; ACS, acetyl-CoA synthase; FAS1 and FAS2, fatty acid synthase; ACC1, acetyl-CoA carboxylase; HMG, HMG-CoA reductase; Erg10, acetoacetyl-CoA thiolase; Erg12, mevalonate kinase; Erg8, phosphomevalonate kinase; MVD, mevalonate pyrophosphate decarboxylase; Erg20, farnesyl pyrophosphate synthetase; GPS, geranyl pyrophosphate synthase; SQS, squalene synthase.

**Fig. 2:**
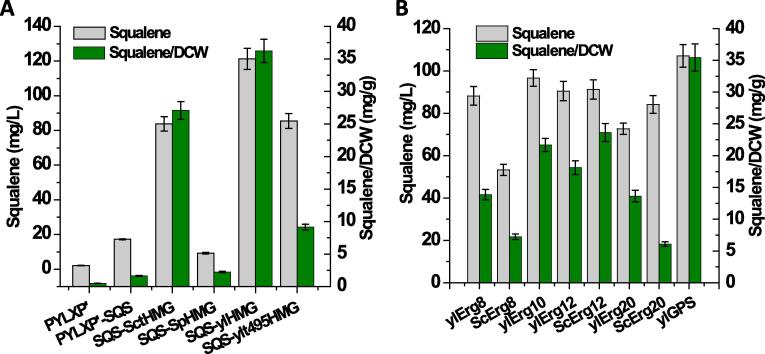
Comparison of different HMG-CoA reductase and identification of rate-limiting steps of endogenous mevalonate pathway in *Y. lipolytica*.

In yeast, squalene was primarily synthesized from the mevalonate (MVA) pathway (Fig. 1). Staring with acetyl-CoA condensation, yeast uses a number of critical enzymes to synthesize squalene, including acetoacetyl-CoA thiolase (Erg10, YALI0B08536g), HMG-CoA reductase (YALI0E04807g), mevalonate kinase (Erg12, YALI0B16038g), phosphomevalonate kinase (Erg8, YALI0E06193g), mevalonate pyrophosphate decarboxylase (MVD1, YALI0F05632g), farnesyl pyrophosphate synthase (Erg20, YALI0E05753g), geranyl pyrophosphate synthase (YALI0D17050g) and squalene synthase (SQS1, YALI0A10076g). Genome annotation indicates that *Y. lipolytica* contains the complete mevalonate pathway (Fig. 1). In MVA pathway, HMG-CoA reductase was reported as the rate-limiting metabolic step in squalene accumulation. In addition, there was almost no squalene accumulated by native *Y. lipolytica* due to the quick consumption of squalene by downstream ergosterol synthase. After we overexpressed the endogenous squalene synthase gene (*SQS*), squalene production was increased to 17.25 mg/L at 120 h with chemically-defined complete synthetic media (CSM-leu) in test tube. With this as a starting strain, the effect of three HMG-CoA reductases (encoded by HMG1) on squalene production was investigated (Rodwell et al., 1976). The three HMG1s were derived from *Saccharomyces cerevisiae*, *Silicibacter pomeroyi* and *Y. lipolytica*. Truncated form of HMG-CoA reductase devoid of N-terminal membrane targeting signal has been proven to be effective in improving isoprenoid production in *Saccharomyces cerevisiae* (encoded by *SctHMG1*) (Huang et al., 2018, Kildegaard et al., 2017). When co-expressed with endogenous SQS, the strain with the truncated HMG1 (SctHMG1) led to squalene production at 83.76 mg/L (Fig. 2A), indicating that overexpression of HMG-CoA reductase was beneficial for squalene production. To test whether other sources of HMG-CoA reductase could display better functions, *SpHMG1* from *Silicibacter pomeroyi* and endogenous *ylHMG1* were co-expressed with SQS, respectively. A low yield of squalene (9.24 mg/L) was produced in the strain expressing *SpHMG1*. This result was consistent with previous findings that HMG1 from *Silicibacter pomeroyi* was highly specific for NADH (Meadows et al., 2016) and this bacterial-derived enzyme could not be directly translated to yeast system. When endogenous *ylHMG1* was co-expressed with SQS (strain HLYaliS01), the engineered strain yielded 121.31 mg/L squalene at 120 h in test tube, demonstrating the potential of using *Y. lipolytica* as a platform to synthesize various terpenes. The truncated form of *ylHMG1* sequence (YALI0E04807p) was also tested, of which the first 495 nucleotides that encode the 165 amino acid N-terminal domain responsible for membrane localization (ER targeting) were removed. The remaining C-terminal residues containing the catalytic domain and an NADPH-binding region (Gao et al., 2017) were overexpressed. The truncated *ylHMG1* (t495*ylHMG1*) was then overexpressed to compare how the variation of *ylHMG1* may improve squalene synthesis. Contrary to our hypothesis, removal of the N-terminal 495 bp of *ylHMG1* exhibits adverse effect on both squalene production and cell growth (Fig. 2A), indicating that the N-terminal membrane-binding domain plays a critical role in squalene synthesis.

In addition to the overexpression of the endogenous *SQS* and *ylHMG1* genes, we also tested whether the expression of other genes in the MVA pathway would improve squalene production, including *ylErg8* encoding phosphomevalonate kinase, *ylErg10* encoding acetoacetyl-CoA thiolase, *Erg12* encoding mevalonate kinase, and *ylErg20* encoding farnesyl pyrophosphate synthetase; *ylGPS* encoding geranyl pyrophosphate synthase. And *ylErg8*, *ylErg10*, *Erg12* and *ylErg20* from *S. cerevisiae* were also overexpressed to compare how the variation of these genes may enhance squalene synthesis. As shown in Fig. 2B, co-overexpression of *ylErg8*, *ylErg10*, *Erg12* could not further improve squalene synthesis, regardless of the source of the gene. Among all of these combinations (Fig. 2B), the highest squalene production was obtained for the strain in which *ylGPS* and *SQS-ylHMG1* were overexpressed, with titer of 107.08 mg/L and a specific production of 36.24 mg/g DCW, which is still lower than the strain only expressing *SQS-ylHMG1*. These results indicate that sequential overexpression of the genes involved in the MVA pathway could not further improve the carbon flux toward squalene, possibly due to the stringent regulation of MVA pathway at multiple nodes, including ergosterol-mediated feedback inhibition or SREBP-related transcriptional repression.

### 3.2 Augmenting NAPDH and acetyl-CoA precursor pathways to improve squalene production

**Fig. 3:**
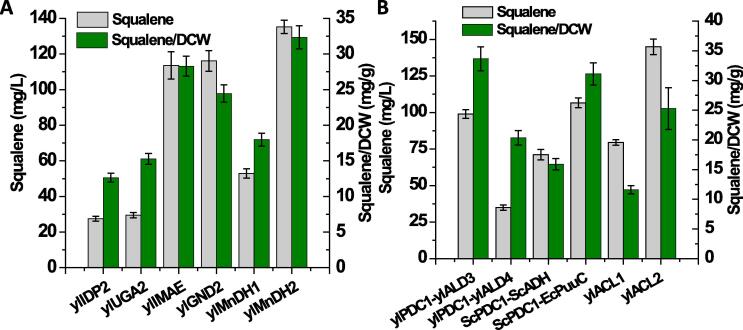
Enhancement of NAPDH and acetyl-CoA precursor pathways to improve squalene production. ScPDC1, pyruvate decarboxylase from *S. cerevisiae*; EcPuuC, aldehyde dehydrogenase from *E. coli*. Other genes are native genes from *Y. lipolytica* and detailed gene annotation could be found in Fig. 1.

NADPH as the primary biological reducing equivalent protects cell from oxidative stress and extend carbon–carbon backbones, which was also reported as the major rate-limiting precursor in fatty acids synthesis in oleaginous species (Qiao et al., 2017). HMG-CoA reductase (HMG1) is the first rate-limiting enzyme in the mevalonate pathway and plays critical role in regulating squalene biosynthesis (Ma et al., 2019). HMG-CoA is reductively hydrolyzed to mevalonate by releasing coenzyme A with NADPH as reducing equivalent (Cao et al., 2017). Based on previous work, source of cytosolic NADPH in the Baker’s yeast may originate from various alternative routes depending on the carbon source and genetic background of the yeast strain (Kavscek et al., 2015, Liu et al., 2019b; S & U, 2011). With glucose as carbon sources, cytosolic NADPH primarily relies on the pentose phosphate pathway. Other cytosolic NADPH pathways include NADP-specific isocitrate dehydrogenase (IDP2), malic enzyme (ylMAE), mannitol dehydrogenase (ylMnDH1, ylMnDH2), 6-phosphogluconate dehydrogenase (ylGND2) and succinate semialdehyde dehydrogenase (ylUGA2) (Liu et al., 2019b) (Fig. 1). In this work, a collection of auxiliary cytosolic NADPH pathways was tested and investigated how these pathways may enhance squalene production and cellular fitness on the basis of co-expression *SQS-ylHMG1* (Fig. 3A). Among these chosen NADPHs, mannitol dehydrogenase (ylMnDH2, encoded by YALI0D18964g) presented the best results to improve squalene production. Mannitol, a more reduced sugar alcohol compared to glucose, played an essential role in modulating cytosolic NADPHs through the mannitol cycle. This could partially explain why mannitol was the major byproduct during lipid accumulation phase in *Y. lipolytica* (Xu et al., 2017b)*.* When ylMnDH2 was overexpressed with SQS and ylHMG1 (strain HLYaliS02, Table 1), the engineered strain produced 11% (0.05 > P > 0.01) more squalene with volumetric production titer increased to 135.22 mg/L, despite relatively decreased yield of 32.33 mg/g DCW (Fig. 3A). This is possibly ascribed to the increased cell fitness and lipid content after enhancing the supplement of NADPH.

Apart from NADPH, acetyl-CoA, is an essential metabolic intermediate connecting glycolysis, Krebs cycle, and glyoxylate shunt pathways. Acetyl-CoA is also the intermediate metabolite participated in lipid synthesis, peroxisomal lipid oxidation and amino acid degradation pathways. It links both anabolism and catabolism, and is the starting molecule in MVA pathway. Cytosolic acetyl-CoA was found as a critical precursor to boost secondary metabolite production (Liu et al., 2019c). For example, engineering alternative cytosolic acetyl-CoA pathways were proven to be efficient strategies to improve fatty acids and isoprenoid production in both Bakers’ yeast and *Y. lipolytica* (Liu et al., 2019a)*.* Therefore, we next investigated whether endogenous and various heterologous acetyl-CoA pathways could improve squalene production. First, the pyruvate decarboxylase (PDC), acetylaldehyde dehydrogenase (ALD) and acetyl-CoA synthase (ACS) bypass (Fig. 1) were investigated and compared the efficiency of this route from *Y. lipolytica*, *S. cerevisiae* and *E. coli* (Fig. 3B). By overexpression of pyruvate decarboxylase (ScPDC) from *S. cerevisiae* and acetylaldehyde dehydrogenase (EcPuuC) from *E. coli*, only 106.54 mg/L of squalene was obtained (Fig. 3B). It was observed that the cell growth fitness was negatively impacted due to the expression of heterologous genes, possibly due to the accumulation of the toxic aldehyde intermediate. The endogenous ATP citrate lyase was subsequently attempted, which is the primary acetyl-CoA route to *Y. lipolytica* metabolism. ATP citrate lyase (ACL) was mainly used for supply of the cytosolic acetyl-CoA. ACL was proven to have two isoforms encoded by two separate genes in *Y. lipolytica* (ACL1 and ACL2) (Nowrousian et al., 2000). Endogenous ylACL1 (YALI0E34793g) and ylACL2 (YALI0D24431g) genes were subsequently tested. A 19.5% (0.05 > P > 0.01) increase in squalene synthesis was obtained in the resulting strains HLYaliS03 with ylACL2 overexpressed along with SQS and ylHMG1, leading to the titer of squalene 144.96 mg/L (Fig. 3B). The increase was probably a result of the pushing strategies for acetyl-CoA enrichment by expressing ACL2 so that adequate cytosolic acetyl-CoA could be pushed into the MVA pathway for the synthesis of squalene (Huang et al., 2018, Jin et al., 2019). Surprisingly, the specific yield reduced to 25.27 mg/g DCW which can be attributed to the increased cell growth and lipid accumulation. This increased lipid content may also serve as the storage space to sequestrate squalene in our engineered cell.

### 3.3 Glucose and acetate as media for squalene production

*Y. lipolytica* can grow on a broad range of substrates and convert various organic wastes to high-value chemicals (Dobrowolski et al., 2016, Rakicka et al., 2017). For example, it has been reported that *Y. lipolytica* possessed strong acetate utilization pathway that is equivalent or even superior to the hexose utilization pathway, which led to an improvement of triacylglyceride (TAG) production from 100 g/L to 115 g/L in bench-top bioreactors, when the cultivation was switched from glucose media to acetate media (Qiao et al., 2017, Xu et al., 2017a). In another work, *Y. lipolytica* was reported to efficiently uptake acetic acid as sole carbon source to produce polyketides up to 4.76 g/L, indicating that acetate may serve as a metabolic “shortcut” to acetyl-CoA with improved carbon conversion efficiency and pathway yield (Liu et al., 2019c). In this study, a similar strategy was explored to investigate the conversion process of acetate to squalene by the engineered strain HLYaliS01, HLYaliS02 and HLYaliS03 (Supplementary data). 41 g/L sodium acetate (NaAc), equivalently to 29.5 g/L acetic acid (HAc, 0.5 M) was used to cultivate the engineered strains. *In situ* pH indicator (bromocresol purple) was used to track the pH change and 6 M HCl was used to adjust the pH in the shake flask. Among the engineered strains, the highest squalene titer reached 191.68 mg/L at 168 h in acetate-YNB medium by strain HLYaliS01, with 99% of acetic acid depleted and 6.6 g/L biomass produced, yielding of squalene at 29.04 mg/g DCW (Supplementary data and Table 2). Strain HLYaliS02 produced 180.28 mg/L squalene at 140 h in acetate-YNB medium with the highest productivity (Supplementary data). When both engineered strains (HLYaliS01 and HLYaliS02) were cultivated in glucose-YNB medium, 157.81 mg/L and 188.18 mg/L squalene was achieved by strain HLYaliS01 and HLYaliS02, with a yield of 16.53 mg/g DCW and 15.91 mg/g DCW, respectively (Supplementary data and Table 2). This indicates that the mannitol cycle (which is engineered in strain HLYaliS02 with *SQS-ylHMG1-ylMnDH2*) may function well when glucose is used as carbon source. Compared with HLYaliS01 and HLYaliS02, HLYaliS03 (the strain with *SQS-ylHMG1-ylACL2*) produced less squalene on glucose (138.33 mg/L), but similar amount of squalene on acetate (176.8 mg/L) (Supplementary data). The data demonstrated that both glucose and acetate could be utilized as carbon sources to produced squalene by *Y. lipolytica* and acetate as a potential and cheap industrial chemical has a promising application and commercial value for squalene and terpene production.

**Table 2:** Comparison of squalene production among modified strains.

Strains	Medium	Squalene productivity (mg/L)	DCW (g/L)	Squalene to DCW yield (mg/g)	Squalene to glucose/acetate yield (mg/g)	Space-time yield (mg/L h)
*HLYaliS01*	Glucose YNB medium	157.81 ± 7.89	9.55 ± 0.48	16.53	3.95	1.09
*HLYaliS02*		188.18 ± 9.21	11.83 ± 0.59	15.91	4.70	0.98
*HLYaliS03*		138.33 ± 5.21	8.91 ± 0.45	15.53	3.46	0.96
*HLYaliS04*		153.30 ± 6.21	15.94 ± 0.55	9.62	3.83	0.80
*HLYaliS01*	Acetate sodium YNB medium	191.68 ± 9.58	6.59 ± 0.33	29.04	6.50	1.14
*HLYaliS02*		180.28 ± 8.89	6.16 ± 0.31	29.27	6.11	1.25
*HLYaliS03*		176.8 ± 6.84	5.00 ± 0.21	35.36	5.99	0.92
*HLYaliS04*		69.62 ± 2.61	4.37 ± 0.23	15.93	2.36	0.36
*HLYaliS02*	Glucose YNB-PBS medium	354.44 ± 16.63	13.98 ± 0.58	25.35	8.86	2.11
*HLYaliS02*	Glucose YNB-PBS medium with cerulenin added	384.13 ± 16.89	14.9 ± 0.62	25.78	9.60	2.00
*HLYaliS02*	C/N ratio 40:1 glucose YNB-PBS medium with cerulenin added	502.75 ± 19.98	15.42 ± 0.76	32.60	12.57	4.19

To further improve squalene synthesis, ylACL2 was assembled to the plasmid harboring SQS, ylHMG1 and ylMnDH2. But the engineered strain (HLYaliS04) did not result in an improved squalene production from either glucose or acetate as substrate, possibly due to the metabolic imbalance or gene expression overloading causing burdensome effects to the cell factory.

### 3.4 Shake flask cultivation of engineered strain with pH and carbon/nitrogen ratio optimization

When glucose was used as the preferred carbon source for cell growth, a similar level of squalene production was detected in the engineered strains (HLYaliS01 and HLYaliS02, HLYaliS03). *Y. lipolytica* is a natural lipid producer, engineered cell could accumulate up to 30%~60% cell weight as lipid, which leads to a strong competition for the precursor acetyl-CoA (Xu et al., 2017b). Meanwhile, cultivation pH and media C/N ratio were two critical factors that affect cellular morphology and growth in *Y. lipolytica* (Szabo, 1999).

**Fig. 4:**
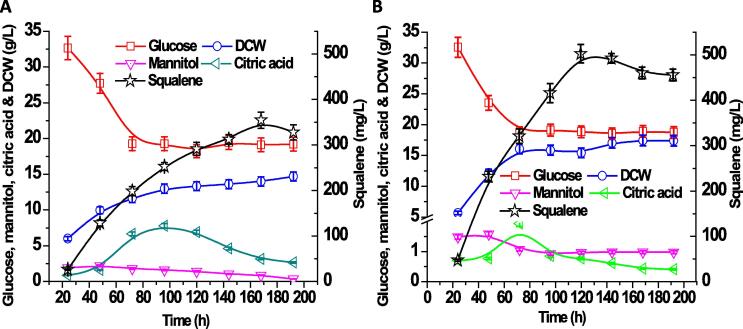
Improving squalene production by controlling pH and C/N ratio with cerulenin supplementation in glucose-minimal media. Fermentation profile of glucose consumption, mannitol, dry cell weight, citric acid and squalene accumulation for strain HLYaliS02 cultivated in glucose-minimal media conditioned with PBS buffer and supplemented with 1 mg/L cerulenin (A). Fermentation profile of glucose consumption, mannitol, dry cell weight, citric acid and squalene accumulation for strain HLYaliS02 cultivated in glucose-minimal media conditioned with PBS buffer, supplemented with 1 mg/L cerulenin and C/N ratio 40:1 (B).

In our previous work, a quick declining of cultivation pH from 6 to 3.5 in polyketide synthesis was observed, due to the accumulation of citric acid when glucose was utilized. The pH variations negatively affect strain physiology, alter cell membrane permeability and limit nutrient transport due to the loss of proton driving force. A significant improvement of polyketide titer was observed by combining PBS buffer with 1 mg/L cerulenin supplementations. Cerulenin is known to irreversibly form a covalent adduct with the active site (cysteine residue) of β-ketoacyl-ACP synthase, inhibiting the elongation of the fatty acid backbone (Liu et al., 2019c). Thus, a similar strategy was applied to promote squalene production by strain HLYaliS02 (shown in Fig. 4 A and Supplementary data). When the engineered strain was cultivated in the minimal YNB media with 0.2 M phosphoric buffer solution (PBS, pH 6.0), squalene production was increased to 354.44 mg/L at 168 h (Supplementary data), which was an increase of 88.4% (0.05 > P > 0.01), compared with the results from pH uncontrolled experiment. The improvement is ascribed to the better growth fitness under pH control and the biomass of strain HLYaliS02 reached 13.98 g/L DCW with the squalene specific yield at 25.35 mg/g DCW (Table 2). The major byproduct mannitol accumulated up to 2.2 g/L at 48 h and citric acid reached 7.81 g/L at 96 h; both mannitol and citrate were subsequently reincorporated into cell metabolism (Supplementary data). But only ~ 50% of glucose was utilized in this process which was consistent with our previous work, indicating the supplementation of PO_4_^3-^ buffer may negatively impact the glucose uptake rate. To further enhance squalene synthesis, 1 mg/L cerulenin was supplemented to the minimal YNB-PBS media at 48 h and the squalene production increased to 384.13 mg/L at 188 h, an 8.4% (0.05 > P > 0.01) increase compared with the result without cerulenin (Fig. 4 A). A similar fermentation profile of glucose consumption, mannitol, and citric acid accumulation was found: half of glucose was utilized while 14.9 g/L DCW was obtained with the squalene specific yield at 25.78 mg/g DCW (Table 2). Byproduct mannitol reached 1.9 g/L at 48 h, but citric acid increased to 9.7 g/L, which was higher than that in the YNB-PBS media without cerulenin supplemented, possibly due to the fact that inhibition of the endogenous fatty acid synthase may prevent citrate from being converted to acetyl-CoA and oxaloacetate by ATP-citrate lyase (encoded by ACL).

We next investigated the effect of C/N ratio on squalene production in YNB-PBS media supplemented with cerulenin (Fig. 4 B). Various C/N ratios including 10:1, 20:1, 40:1, 60:1 and 80:1 was studied (Supplementary data). When the C/N ratio was set at 60:1, a similar fermentation profile of glucose consumption, mannitol, dry cell weight, citric acid was obtained, compared to the metabolic profile for C/N 80:1. Squalene titer at C/N 60:1 reached 396 mg/L at 120 h with increased productivity. The highest squalene titer was achieved in the media with C/N ratio 40:1, reaching 502.75 mg/L at 120 h with the yield to 32.6 mg/g DCW (Fig. 4 B, Table 2), which was 30.8% higher than the squalene production form C/N ratio 80:1 media. It was speculated that acetyl-CoA flux was enlarged and flowed to MVA pathway, since less citric acid accumulation (1.9 g/L) was observed at the end of fermentation. However, when the C/N ratio was further reduced to 20:1 or 10:1, adverse effect was obtained with decreasing squalene production (Supplementary data). It was speculated that the superfluous nitrogen provision may partition more carbon to cell growth. These results illustrated that C/N ratio plays an important role in the redistribution of carbon flux and strongly influenced the accumulation of squalene. Further downregulation of acetyl-CoA carboxylase (ACC) may be required to improve squalene production. ACCase, as the malonyl-CoA source pathway and the acetyl-CoA sink pathway during lipogenesis, was primarily controlled through the phosphorylation of serine residues by Snf1-mediated AMP kinase. Inhibition of fatty acid synthase pathway and nitrogen starvation was proven to be effective strategies to activate Snf1 kinase and slows down ACC1 activity in *Y. lipolytica* (Seip et al., 2013, Zhang et al., 2013). It was consistent with the findings that medium C/N ratio was beneficial for squalene synthesis. By applying these engineering strategies, an oleaginous yeast strain with a high squalene production was obtained. This work highlights the potential of engineering *Y. lipolytica* as a promising microbial platform for efficient synthesis of squalene and terpene-related compounds.

## 4 Conclusion

In this work, *Yarrowia lipolytica* was used as the microbial host for squalene production. Upon overexpression of squalene synthase, HMG-CoA reductase, mannitol dehydrogenase or ATP-citrate lyase, and the cultivation conditions, 502.7 mg/L squalene was obtained in shake flaks with C/N 40:1 media conditioned with PBS buffer with supplementation of 1 mg/L cerulenin. It was demonstrated that the native squalene pathway of *Y. lipolytica* could be harnessed as an efficient metabolic route to synthesize squalene. This work may serve as a starting point to harness *Y. lipolytica* as an oleaginous yeast factory for cost-efficient production of squalene or terpene-based chemicals.

## CRediT authorship contribution statement

**Huan Liu:** Data curation, Investigation, Methodology, Writing - original draft. **Fang Wang:** . **Li Deng:** . **Peng Xu:** Conceptualization, Funding acquisition, Investigation, Project administration, Supervision, Writing - review & editing.

## Declaration of Competing Interest

The authors declare that they have no known competing financial interests or personal relationships that could have appeared to influence the work reported in this paper.
