# Supplementary material for: Genetic and bioprocess engineering to improve squalene production in Yarrowia lipolytica
Source: Bioresour Technol. 2020 Dec;317:123991. doi: 10.1016/j.biortech.2020.123991 (PMC7561614; doi:10.1016/j.biortech.2020.123991)
Supplement: Supplementary Data 1 [file mmc1.docx]

*Supplementary files*

**Genetic and bioprocess engineering to improve squalene production in *Yarrowia lipolytica***

Huan Liu^1,2^, Fang Wang^2^, Li Deng^2,^^[[1]](#footnote-1)^Φ and Peng Xu^1^^[[2]](#footnote-2)^*

^1^Department of Chemical, Biochemical and Environmental Engineering, University of Maryland, Baltimore County, Baltimore, MD 21250.

^2^College of Life Science and Technology, Beijing University of Chemical Technology, Beijing, China.


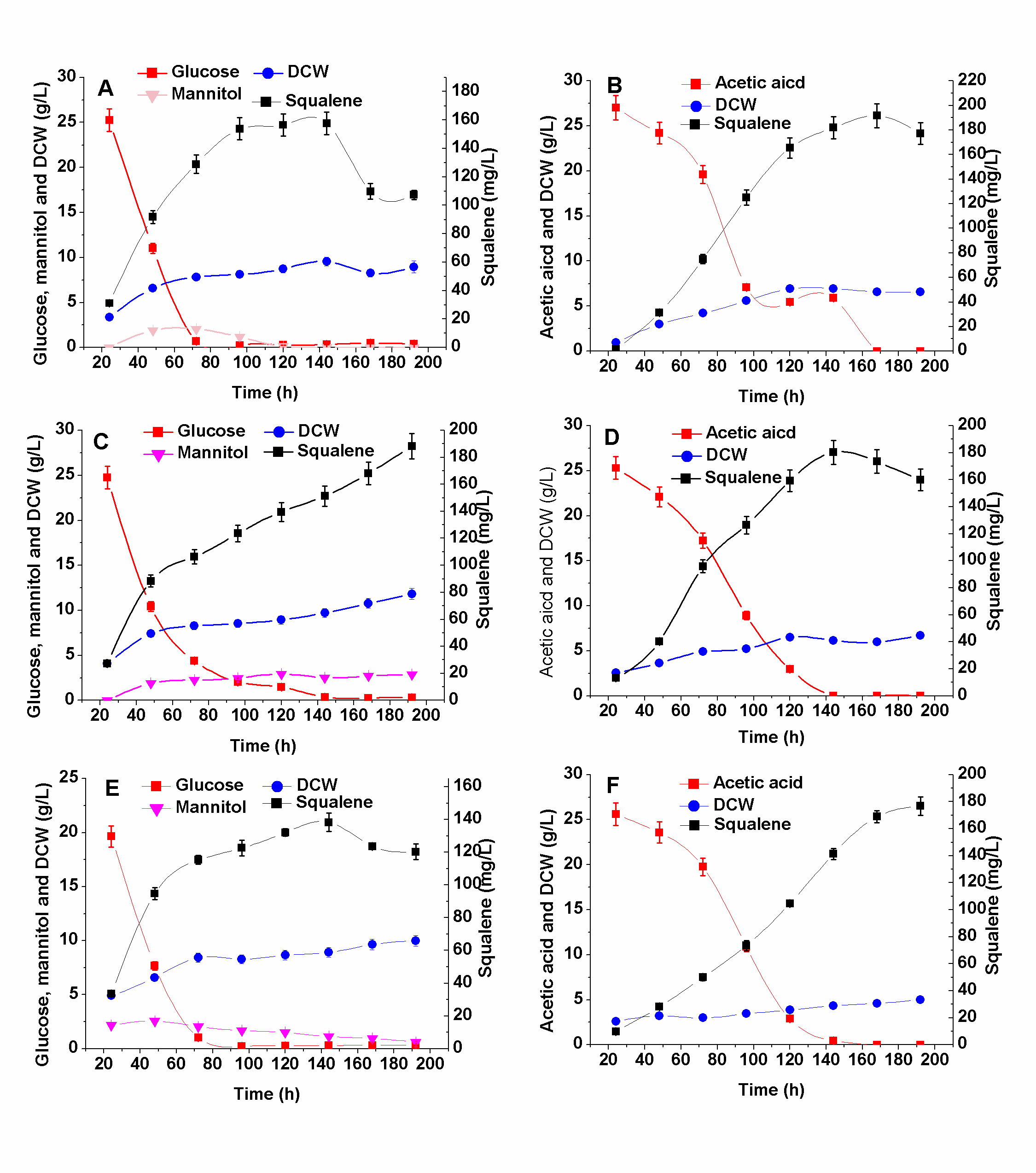


Supplementary Fig. S1 Comparison of squalene production using glucose and acetate as substrate by different engineered strans. Squalene production from minimal media supplemented with glucose and NaAc by strain *HLYaliS01* (A and B); Squalene production from minimal media supplemented with glucose and NaAc by strain *HLYaliS02* (C and D); Squalene production from minimal media supplemented with glucose and NaAc by strain *HLYaliS03* (E and F). When NaAc was used as substrate, the medium pH will increase with the consumption of acetate. We adjusted the pH to 6.0 by adding HCl. Bromocresol purple is a pH-sensitive indicator to track the pH variations of fermentation process.





Supplementary Fig. S2 Comparison of squalene production using glucose and acetate as substrate by *HLYaliS04.* Squalene production from minimal media supplemented with glucose by strain *HLYaliS04* (A); Squalene production from minimal media supplemented with NaAc by strain *HLYaliS04* (B).


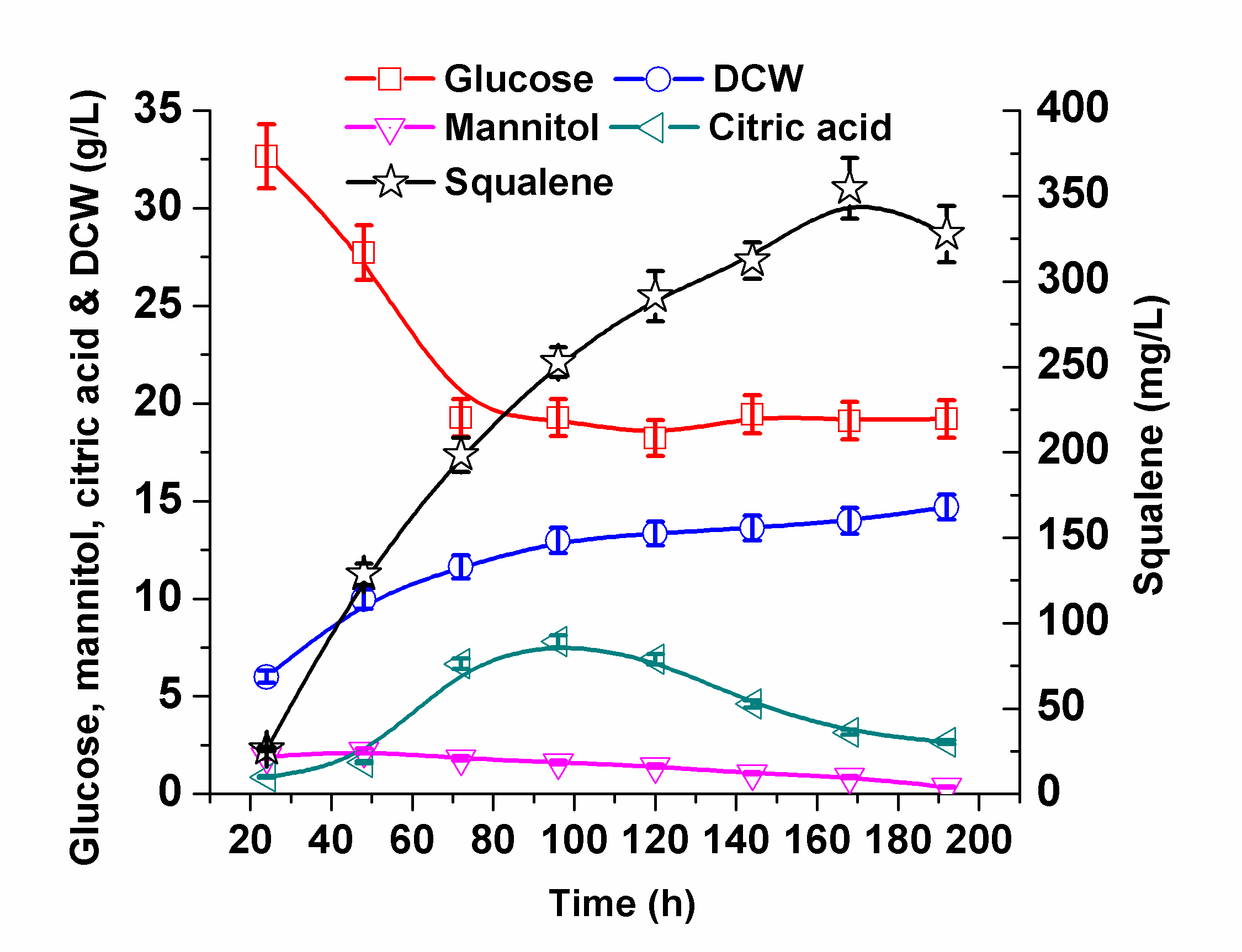


Supplementary Fig. S3 The effect of pH control on the production of squalene by strain *HLYaliS02*. Fermentation profile of glucose consumption, mannitol, dry cell weight, citric acid and squalene accumulation for strain *HLYaliS02* cultivated in glucose-minimal media conditioned with PBS buffer.





Supplementary Fig. S4 The effect of C/N ratio on the production of squalene by strain *HLYaliS02.* Fermentation profile of glucose consumption, mannitol, dry cell weight, citric acid and squalene accumulation for strain *HLYaliS02* cultivated in glucose-minimal media conditioned with PBS buffer, supplemented with 1 mg/L cerulenin and C/N ratio 10:1 (A), 20:1 (B), 40:1 (C), 60:1 (D).

**Supplementary Table S1. Primers and synthetic oligos/genes used in this study**

| **No.** | **Primer** | **Nucleotide sequence (5’ >3’)** |
| --- | --- | --- |
| 1 | SQS_F | ccgaccagcactttttgcagtactaaccgcagggaaaactcatcgaactgctcttgc |
| 2 | SQS_R | ggggacaggccatggaactagtcggtaccctaatctctcagaggaaacatc |
| 3 | ylHMG_F | ccgaccagcactttttgcagtactaaccgcagctacaagcagctattggaaagattg |
| 4 | ylHMG_R | ggggacaggccatggaactagtcggtaccctatgaccgtatgcaaatattcg |
| 5 | ylt495HMG_F | ccgaccagcactttttgcagtactaaccgcagctacgagaagttgtgcgaaccc |
| 6 | ylt495HMG_R | ggggacaggccatggaactagtcggtaccctatgaccgtatgcaaatattcg |
| 7 | SpHMG_F | ccgaccagcactttttgcagtactaaccgcagacagggaaaaccggccatatag |
| 8 | SpHMG_R | ggggacaggccatggaactagtcggtacctcatgtattttccagaacctgc |
| 9 | SctHMG_F | ccgaccagcactttttgcagtactaaccgcaggaccagcttgttaaaacagaag |
| 10 | SctHMG_R | ggggacaggccatggaactagtcggtaccttatgattttatgcaggttactg |
| 11 | ylErg8_F | ccgaccagcactttttgcagtactaaccgcagaccacctattcggctccggg |
| 12 | ylErg8_R | ggggacaggccatggaactagtcggtaccctacttgaaccccttctcgagc |
| 13 | ylErg10_F | ccgaccagcactttttgcagtactaaccgcagcgactcactctgccccgacttaacg |
| 14 | ylErg10_R | ggggacaggccatggaactagtcggtaccctacttgaaccccttctcgagcc |
| 15 | ylErg12_F | ccgaccagcactttttgcagtactaaccgcaggactacatcatttcggcgccaggc |
| 16 | ylErg12_R | ggggacaggccatggaactagtcggtaccctaatgggtccagggaccgatg |
| 17 | ylErg20_F | ccgaccagcactttttgcagtactaaccgcagtccaaggcgaaattcgaaagcgtg |
| 18 | ylErg20_R | ggggacaggccatggaactagtcggtaccctacttctgtcgcttgtaaatc |
| 19 | ScErg8_F | ccgaccagcactttttgcagtactaaccgcagtcagagttgagagccttcagtgcc |
| 20 | ScErg8_R | ggggacaggccatggaactagtcggtaccttatttatcaagataagtttcc |
| 21 | ScErg12_F | ccgaccagcactttttgcagtactaaccgcagtcattaccgttcttaacttctgc |
| 22 | ScErg12_R | ggggacaggccatggaactagtcggtaccttatgaagtccatggtaaattcg |
| 23 | ScErg20_F | ccgaccagcactttttgcagtactaaccgcaggcttcagaaaaagaaattaggagag |
| 24 | ScErg20_R | ggggacaggccatggaactagtcggtaccctatttgcttctcttgtaaac |
| 25 | ylGPS_F | ccgaccagcactttttgcagtactaaccgcaggattataacagcgcggatttcaagg |
| 26 | ylGPS_R | ggggacaggccatggaactagtcggtacctcactgcgcatcctcaaagtac |
| 27 | ylMAE _F | ccgaccagcactttttgcagtactaaccgcagttacgactacgaaccatgcgaccc |
| 28 | ylMAE _R | ggacaggccatggaactagtcggtaccctagtcgtaatcccgcacatggatg |
| 29 | ylMnDH1 _F | ccgaccagcactttttgcagtactaaccgcagcctgcaccagcaacctacgctactg |
| 30 | ylMnDH1 _R | ggacaggccatggaactagtcggtacctcaaggacaacagtagccgccatc |
| 21 | ylMnDH2 _F | ccgaccagcactttttgcagtactaaccgcagtctggaccttccaccctcgccacg |
| 32 | ylMnDH2 _R | ggacaggccatggaactagtcggtacctcagaggcaaggtagaggtaggtag |
| 33 | ylIDP2 _F | ccgaccagcactttttgcagtactaaccgcagtccaccaccgctactcgaggcctg |
| 34 | ylIDP2 _R | ggacaggccatggaactagtcggtaccctaagccaggtccttcttcagtc |
| 35 | ylGND2_F | ccgaccagcactttttgcagtactaaccgcagactgacacttcaaacatcaagtg |
| 36 | ylGND2_R | ggacaggccatggaactagtcggtaccttaagcatcgtaagtggaagaag |
| 37 | ylUGA2 _F | ccgaccagcactttttgcagtactaaccgcagttgcgagccctgaataccgtccag |
| 38 | ylUGA2 _R | ggacaggccatggaactagtcggtaccttaaggctgaatgtggggctcgacg |
| 39 | ylPDC1 _F | ccgaccagcactttttgcagtactaaccgcagagcgactccgaaccccaaatggtc |
| 40 | ylPDC1_R | ggacaggccatggaactagtcggtaccctaaacgttggtcttggcagagag |
| 41 | ylALD4 _F | ccgaccagcactttttgcagtactaaccgcagtctcttttcagcaaacttaccctag |
| 42 | ylALD4_R | ggacaggccatggaactagtcggtaccttactgcagcttggcctggtcaaac |
| 43 | ylALD3 _F | ccgaccagcactttttgcagtactaaccgcagcaagttactcttcccgacggaaag |
| 44 | ylALD3_R | ggggacaggccatggaactagtcggtaccctaatccaggttaatgtggac |
| 45 | ylACL1 _F | ccgaccagcactttttgcagtactaaccgcagtctgccaacgagaacatctcccg |
| 46 | ylACL1_R | ggggacaggccatggaactagtcggtaccctatgatcgagtcttggccttgg |
| 47 | ylACL2 _F | ccgaccagcactttttgcagtactaaccgcagtcagcgaaatccattcacgaggc |
| 48 | ylACL2_R | ggggacaggccatggaactagtcggtaccttaaactccgagaggagtgg |
| 49 | ScPDC1_F | ccgaccagcactttttgcagtactaaccgcagtctgaaattactttgggtaaatatttg |
| 50 | ScPDC1_R | ggggacaggccatggaactagtcggtaccttattgcttagcgttggtagc |
| 51 | ScADH_F | ccgaccagcactttttgcagtactaaccgcagtctatcccagaaactcaaaaagg |
| 52 | ScADH_R | ggggacaggccatggaactagtcggtaccttatttagaagtgtcaacaacg |
| 53 | EcPuuc_F | ccgaccagcactttttgcagtactaaccgcagaattttcatcatctggcttactggc |
| 54 | EcPuuc_R | ggggacaggccatggaactagtcggtacctcaggcctccaggcttatccag |

1. Φ Corresponding author Tel: +86-010-64414543; fax: +86-010-64416428.

   E-mail address: dengli@mail.buct.edu.cn (Li Deng). [↑](#footnote-ref-1)
2. * Corresponding author Tel: +1(410)-455-2474; fax: +1(410)-455-1049. E-mail address: pengxu@umbc.edu (PX). [↑](#footnote-ref-2)
